# NaCl-Supplemented Alfalfa-Based TMR Improves Meat Quality by Enhancing Essential Amino Acids and Optimizing Fatty Acid Profile in AOHU Sheep Under Simulated Saline–Alkaline Conditions

**DOI:** 10.3390/foods14244206

**Published:** 2025-12-07

**Authors:** Hunegnaw Abebe, Ruochen Yang, Guicong Wei, Jiajun Cui, Haixin Wang, Xiaoran Feng, Mulugeta Walelegne, Junnan Ma, Luxin Kong, Yan Tu

**Affiliations:** 1 Beijing Key Laboratory for Dairy Cow Nutrition, Institute of Feed Research, Chinese Academy of Agricultural Sciences, Beijing 100081, China; 2 Department of Animal Sciences, College of Agriculture, Wollo University, Dessie P.O. Box 1145, Ethiopia; 3 College of Animal Science and Technology, Guangxi University, Nanning 530005, China; 4 College of Animal Science and Technology, Gansu Agricultural University, Lanzhou 730070, China; 5 Ethiopian Institute of Agricultural Research, Holetta Agricultural Research Center, Addis Ababa P.O. Box 2003, Ethiopia

**Keywords:** alfalfa, essential amino acids, fatty acid profile, meat quality, NaCl supplementation, ruminant nutrition, saline–alkaline soil

## Abstract

Saline–alkaline soils are increasingly reducing global forage productivity and may indirectly compromise the nutritional quality of animal-derived foods for human consumption. Alfalfa, grown under saline–alkaline conditions, often accumulates sodium, thereby altering its nutritional composition and digestibility. NaCl was added to an alfalfa-based total mixed ration (TMR) to model saline-alkaline alfalfa with high salt content. This method is a simplified modeling approach wherein sodium chloride is used to simulate saline stress. We have studied, using this model, changes in growth performance, nutrient digestibility, amino acid composition, and meat quality of AOHU (Hu × Australian White) lambs. The levels of dietary NaCl were set at 0.43% (to reflect the baseline NaCl content of standard alfalfa-based TMR and 1.71% (to reflect a level of high-salt alfalfa produced under saline–alkaline growing conditions). Compared to the control group, supplementation with NaCl enhanced the average daily gain, feed conversion efficiency, relative growth rate, and dry matter intake (*p* < 0.05). Apparent digestibility of dry matter, organic matter, crude protein, ether extract, fiber fractions (NDF and ADF), and gross energy was also improved (*p* < 0.001), reflecting enhanced nutrient utilization. Total saturated fatty acids had decreased, while meat redness increased, and the PUFA/SFA ratio increased, reflecting a leaner and healthier lipid profile. Moreover, essential amino acids-threonine, valine, methionine, isoleucine, leucine, and lysine-were significantly higher (*p* < 0.05), revealing a better meat nutritional quality. In summary, dietary NaCl supplementation in an alfalfa-based TMR effectively simulates saline–alkaline conditions that improve growth performance, nutrient digestibility, and meat nutritional composition. Results from this study show how NaCl supplementation in alfalfa-based rations—used here to mimic the elevated salt levels found in alfalfa grown on saline–alkaline soils—affects growth performance, nutrient digestibility, and meat quality in lambs, providing insight for feeding strategies in salt-affected production systems.

## 1. Introduction

With the continuous growth in world demand for high-quality animal protein, especially from ruminants, it is of importance to maximize feed efficiency and meat quality sustainably [1,2]. Harnessing the opportunity for saline–alkaline soils, which are now expanding globally and taking a significant portion of cultivable land [3,4], remains hugely underutilized despite its considerable potential to support forage production in livestock systems. Alfalfa (*Medicago sativa* L.) is highly nutritious and resilient, and has a better tolerance to saline-alkaline soil. Its tolerance of saline-alkaline soils makes it a potential candidate feed crop for marginal land [5,6], yet little research has been conducted into how saline-alkaline stress-induced alteration in alfalfa quality affects animal performance and, in particular, nutrient digestibility, growth performance, meat quality, fatty acid and amino acid profiles. This represents a significant knowledge gap, especially on nutrient digestibility and meat quality in sheep. More than 952 million hectares have been salinized worldwide, with the most salinized regions in Asia, Africa, and Australia, including China, Kazakhstan, and Iran [4]. It is necessary to develop feeding systems that are compatible with the prevailing saline-alkaline conditions to enhance livestock production in such regions.

Alfalfa produced in saline–alkaline conditions may accumulate elevated levels of sodium with other soluble ions like chloride, bicarbonate, carbonate, calcium, and magnesium that could change the nutritional profile and affect animal metabolisms. Although NaCl has been reported to enhance nutrient absorption and water intake in ruminants [7], its direct application in TMR replicating salty forage and effects on meat quality are not well studied. We selected AOHU sheep, a cross between Australian White rams (with high growth and meat yield) [8] and Hu ewes (with fertility and heat tolerance) [9]—as a suitable model for this trial. Their versatility renders them ideal for testing nutritional strategies under stress-vulnerable conditions.

Since naturally produced high-salinity alfalfa is often inconsistent in supply, a controlled NaCl-supplementation model is used to simulate the salt load of saline-affected forage. This permits targeted investigation of sheep responses to salt-associated changes in dietary composition under standardized conditions. A concentration of 1.71% NaCl (DM basis) was chosen because it is within the range of commonly reported salt levels of alfalfa grown on saline–alkaline soils, and thus serves as a realistic high-salinity forage condition [10] and therefore reproduces a realistic “worst-case” herbage scenario without exceeding the 2% total-dietary-salt limit advised by [11]. This approach enabled us to evaluate the specific effects of an alfalfa-based TMR reflecting the composition of forage produced under high-salinity soil conditions on AOHU sheep responses.

The present study builds upon our previous work [12], which investigated the impact of NaCl supplementation of an alfalfa-based TMR on ruminal fermentation characteristics, nutrient utilization, and antioxidant status in AOHU lambs to simulate alfalfa grown under high-salinity soil conditions. Building on these findings, the present research further investigated how NaCl-supplemented alfalfa-based TMR on meat performance, specifically including carcass traits (carcass yield and dressing percentage), amino acid composition, fatty acid profiles, nutrient digestibility, Meat pH, color, drip loss, and shear force that reflect meat quality improvement under simulated saline–alkaline feeding conditions.

This research, by simulating the characteristics of salt-affected forage, investigated whether or not NaCl-supplemented alfalfa-based TMR might provide a feasible approach to supporting sheep productivity and meat nutritional quality across saline-prone regions, with preliminary insights into future research and practical applications.

## 2. Materials and Methods

### 2.1. Experimental Animals and Feeding Management

The experiment was conducted for 90 days (August–October 2024) in indoor pens at the Nankou experimental site, Beijing, China, after approval from the Animal Ethics Committee of the Chinese Academy of Agricultural Sciences (IFR-CAAS-2024-07-22) and following institutional animal care guidelines. Twenty-four weaned male AOHU lambs with ages from 2 to 3 months and not castrated were randomly allotted to two treatments according to their initial body weight as follows: control: 22.64 ± 4.4 kg; NaCl-supplemented: 22.36 ± 4.05 kg in a completely randomized design. Each pen was stocked with four lambs, with each pen having an area of 12 m^2^ and two drinkers. Individual lambs were the experimental unit in all measurements except water intake that was recorded on a group basis.

The control group received alfalfa harvested from non-saline–alkaline soil with NaCl supplemented to 0.43% DM, whereas the treatment group received the same alfalfa-based ration with NaCl supplemented to 1.71% DM, approximating the Na and Cl concentrations found in alfalfa grown on heavily saline–alkaline soils (acknowledging that NaCl supplementation cannot fully simulate the broader ionic composition of saline–alkaline environments (e.g., CO_3_^2−^, HCO_3_^−^, Mg^2+^, Ca^2+^)). The 90-day experiment included a 20-day adaptation period to housing and automatic feeders, a 10-day acclimation to the experimental diets (data excluded), and a 60-day feeding trial during which growth performance, nutrient digestibility, meat yield, and meat quality parameters were measured.

Diets were formulated to meet the established standards for growing lambs [11] (Table 1), and mechanical mixing added water (2 L for every 82.33 kg TMR) to reduce dust and improve cohesion. Feed was stored under controlled conditions for no more than four days to avoid spoilage, and lambs had free access to water and were fed with 85.41% DM TMR twice a day (07:00 and 17:00). Feed intake was automatically recorded with RFID identification by ear tag (YMT-SF-02, Tai’an Yimeite Machinery Co., Ltd., Tai’an, Shandong, China). Lambs were pre-dewormed, clinically healthy at the beginning of the trial, and there were no health issues or deaths throughout the experiment. Barn sanitation once a week was performed using glutaral and deciquam as disinfectants, and all the lambs were kept under similar management conditions throughout the trial period. The experimental design and feeding management followed the protocol described in our previous study [12].

### 2.2. Experimental Feed Chemical Composition Analysis

Dried samples were ground in a Thomas Wiley^®^ Model 4 mill (Thomas Scientific, Swedesboro, NJ, USA) fitted with a 40-mesh screen. Dry matter (DM) was determined by oven-drying the samples at 105 °C to constant weight. Ash content was determined by combustion in a muffle furnace (SX2-4-10, Nanyang Dadi Electric Equipment Co., Ltd., Henan, China) at 550 °C for 4 h [13]. Nitrogen (N) content was analyzed by the Kjeldahl method and converted to crude protein using the factor of 6.25 [13]. Ether extract was determined using petroleum ether in a Soxhlet apparatus (BGZ-DZ, Beijing Zhongxing Weiye Instrument Co., Ltd., Beijing, China). Calcium was quantified by titration following acid digestion, while phosphorus (P) was analyzed using the molybdovanadate colorimetric procedure [13]. Gross energy was determined with a bomb calorimeter and is expressed in MJ/kg. Neutral detergent fiber (NDF) and acid detergent fiber (ADF) were analyzed using the methods of Van Soest et al., with NDF assayed using heat-stable amylase [14]. Sodium in the TMR was determined by flame photometry after acid digestion and expressed as NaCl using the molecular-weight conversion factor Na (%) × 2.54 [12] (based on the relative molecular masses of sodium and chloride).

### 2.3. Feed and Water Intake

Daily dry matter intake (DMI) was recorded for all sheep using an automated feeding system that continuously monitored the amount of alfalfa-based total mixed ration (TMR) offered. The individual DMI per day was precisely determined and was utilized for feed intake and performance indicator calculation. Water was available ad libitum to all lambs from a common trough. water intake was recorded per pen and expressed as pooled values for each treatment group (12 sheep) Water intake was measured at the group level (12 sheep per group) as the change in trough water volume between 07:00 and 17:00 daily. The daily total water intake per group was then divided by group size to estimate the average daily water intake per sheep. Since individual water consumption was not measured, there were no replicates at the biological level, and statistical analysis of these data was not performed. Only mean comparison was performed.

### 2.4. Growth Performance

Growth performance was monitored by recording body weight at the beginning and end of the experimental period. This was consistently performed before morning feeding to avoid discrepancies. Average daily gain (ADG) was calculated as the total weight gain divided by the number of days in the experimental period. Relative growth rate was calculated as the total weight gain divided by the initial weight multiplied by 100 to give a percentage measure of proportional growth. Feed conversion efficiency (FCR), one of the critical indicators of feed efficiency, was computed as the total feed intake divided by the total weight gain.

### 2.5. Apparent Digestibility

At the completion of the trial, six sheep from each treatment group were randomly taken for digestion tests. The digestibility trial lasted for 7 days, with the first two days being an adaptation period. Faces and urine were collected daily using digestive and metabolic cages to ensure the precise measurement and separation of waste products. A proportion of 20% of the fecal samples collected every day were preserved in polyethylene plastic buckets to prevent any contamination. Nutrient apparent digestibility was determined as follows: [(nutrient intake-nutrient excreted)/nutrient intake] × 100%. All feeds were dried at 65 °C for about 48 h by removing the moisture right before the analytical processes, as similarly performed with each fecal sampling. The dried samples were ground using a Willey mill to pass through a 40-mesh screen and allowed to equilibrate at room temperature for 24 h before being sealed in airtight paper bags for further examination. The chemical composition of the samples was assessed for key nutrients: dry matter (DM), crude protein (CP), organic matter (OM), ether extract (EE), gross energy (GE), neutral detergent fiber (NDF), and acid detergent fiber (ADF), according to the previously described method.

### 2.6. Slaughtering, Carcass Traits and Slaughter Performance

At the end of the feeding trial, eight lambs per treatment were randomly selected for slaughter after a 12 h feed fasting with free access to water. Slaughtering was performed in a licensed commercial abattoir in full accordance with national animal welfare standards. The lambs were exsanguinated and then skinned and dressed by qualified personnel. The live weight prior to slaughter was recorded just before exsanguination, and hot carcass weight was recorded just after dressing. Dressing percentage was determined as carcass weight divided by LWBS × 100. Total tissue depth (GR) value was determined on the basis of the measurement 11 cm off the dorsal midline between the 12th and the 13th ribs using a vernier caliper; three measurements per lamb were averaged for analysis.

### 2.7. Meat pH, Color, Drip Loss and Shear Force

Meat quality was assessed using the Longissimus thoracis (LT) muscle. Carcasses were chilled at 4 ± 1 °C for 24 h postmortem. The eye muscle area (EMA) was measured on the chilled carcass by making a transverse cut between the 12th and 13th ribs on the left side. The cross-sectional area of the LT was calculated using the following formula: EMA (cm^2^) = muscle height × muscle width × 0.7 [15]. Muscle pH was determined at 45 min and 24 h postmortem using a portable pH meter with automatic temperature compensation (Testo 206-pH2, Testo, Titisee-Neustadt, Germany). The probe was inserted approximately 2 cm into the LT muscle. The pH meter was calibrated at room temperature using standard buffer solutions (pH 4.00 and 7.00) before each use.

Meat color parameters—lightness (L*), redness (a*), and yellowness (b*)—were measured at 24 h postmortem using a colorimeter (Konica Minolta, Tokyo, Japan). The device used a D65 light source, 10° standard observer angle, and an 8 mm aperture according to the International Commission on Illumination color system (CIE) standard. Before color assessment, samples were allowed to bloom for 30 min at room temperature to ensure sufficient oxygenation. The hue angle was calculated as: Hue angle = tan^−1^(b*/a*). Drip loss was evaluated by cutting the LT muscle into standardized pieces (5 × 3 × 2 cm), weighing them immediately (initial weight), then suspending them in plastic bags at 4 °C for 24 h. Afterward, the samples were reweighed (final weight), and drip loss was calculated as: Drip loss (%) = 100 × (initial weight—final weight)/initial weight.

Warner–Bratzler shear force (WBSF) was used to evaluate meat tenderness. Raw LT muscle was used for WBSF determination because shear force in raw meat is more sensitive to differences in intrinsic muscle structure (for example, connective tissue integrity and muscle fiber characteristics), enabling the detection of treatment-related changes before the confounding effects of cooking that alter protein denaturation and moisture loss. Cylindrical core samples (1.27 cm diameter, 2.54 cm length) were prepared from raw LT muscle using a handheld coring device. The average weight of core specimens was 3.4 ± 0.4 g. Shear force was measured using a C-LM3B shear force device (Tenova, Harbin, China), equipped with a standard V-shaped blade, a high-accuracy load cell (max 500 N), and a crosshead speed of 200 mm/min. Each sample was measured in triplicate under uniform handling conditions.

### 2.8. Meat Amino and Fatty Acid Profiles

Sixteen LT samples (n = 8 per treatment group) were analyzed at the Nanjing Established Institute of Biological Engineering, Beijing. Amino acid profiles were determined following hydrolysis with 6 N HCl, vacuum drying, filtration, and 20-fold dilution in pH 2.2 buffer using a Sykam S-433D automatic amino acid analyzer (Sykam GmbH, Eresing, Bavaria, Germany) under the guidelines of [13]. The equipment was operated at the detection wavelengths of 570 and 440 nm, with the mobile phase as sodium citrate buffer, a flow rate of 0.45 mL/min, and a temperature gradient of 58–74 °C under 30–40 bar of pressure. All fatty Acid (FA) content is expressed as mg/100 g muscle tissue to allow inter-comparison with human diet recommendations. The internal standard used was C13:0 methyl ester, which was added prior to methylation for proper quantitation. The column used was a TG-FAME capillary column (100 m × 0.25 mm × 0.2 μm, Thermo Fisher Scientific (China) Co., Ltd., Shanghai, China), and GC analysis was performed with an Agilent 7890A (Agilent Technologies (China) Co., Ltd., Beijing, China) using FID at 280 °C. The FA analysis followed the standard method [16], and identification/quantification was based on a 37-FAME standard mixture (Sigma-Aldrich) and employed in quantitation with the calculated data as follows, W = (C × V × K × N)/M × 10^−4^.

### 2.9. Data Analysis

Statistical analyses were performed using SPSS (Statistical Package for the Social Sciences, version 28.0, IBM Corp., Armonk, NY, USA) and GraphPad Prism (version 9.5.1, GraphPad Software). Data were assessed for normality using the Shapiro–Wilk test and for homogeneity of variances using Levene’s test. The individual lamb served as the experimental unit for all analyses except water intake, which was recorded on a pen basis and analyzed at the group level. Amino acid profiles, fatty acid composition, and nutrient digestibility were analyzed using one-way ANOVA in SPSS. Where ANOVA revealed significant treatment effects, Tukey’s post hoc test was used for pairwise comparisons. Carcass yield, dressing percentage, drip loss, shear force, eye muscle area, dry matter intake (DMI), water intake, average daily gain (ADG), feed conversion ratio (FCR), body weight change (BWC) and relative growth rate (RGR) were analyzed using two-tailed independent *t*-tests in GraphPad Prism. Repeated-measures ANOVA was performed only for meat pH and color parameters (L*, a*, b*) measured at 0 and 24 h postmortem, using time as within-subject factor and treatment as between-subject factor. Values are expressed as mean ± standard error of the mean (SEM), and statistical significance was declared at *p* < 0.05.

## 3. Results

### 3.1. Intake, Growth Performance, and Feed Efficiency

**Figure 1:**
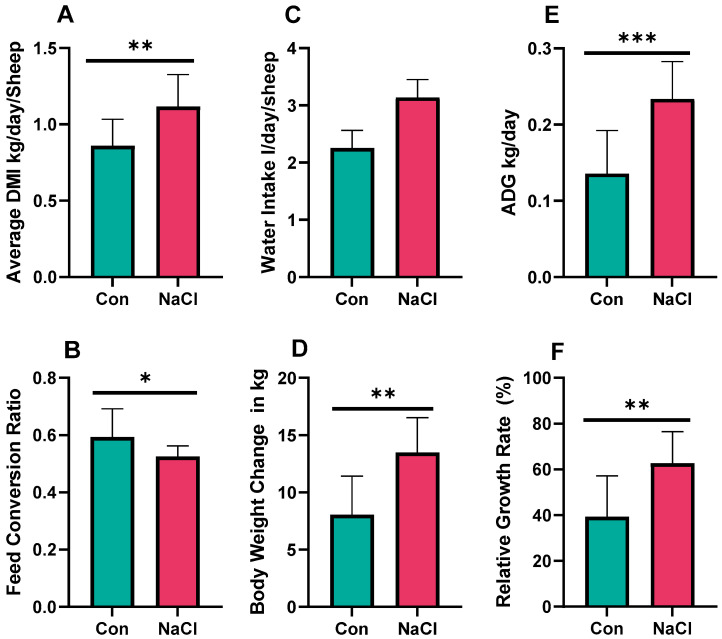
Effect of NaCl supplementation on intake and growth performance of sheep. (**A**) Dry matter intake; (**B**) water intake; (**C**) average daily gain; (**D**) feed conversion ratio; (**E**) body weight change; (**F**) relative growth rate; Con = control; control diet contained 0.43% NaCl (DM basis); NaCl-supplemented diet contained 1.71% NaCl (DM basis); *** *p* < 0.001; ** *p* < 0.01; * *p* < 0.05. Values are expressed as mean ± SEM, n = 12.

The results indicate that AOHU sheep supplemented with NaCl have a high increase in feed intake, growth performance, and water intake (Figure 1). DMI was highly significant in the NaCl-supplemented group compared to the control group (Figure 1A; *p* < 0.001). Water intake, as measured at the group level by monitoring trough volume changes between 07:00 and 17:00 daily, was estimated to be 2.25 L/day/sheep for the control group and 3.14 L/day/sheep for the NaCl group (Figure 1C). Although this trend suggests higher water intake with NaCl supplementation, statistical comparisons were not attempted, due to the absence of biological replication for the water intake measurements. ADG (Figure 1E) and body weight change (Figure 1D) showed increased values at *p* < 0.001 and <0.01, respectively, reflecting improved growth performance. The relative growth rate (Figure 1F) was also higher in the NaCl group, at *p* < 0.01, reflecting better growth efficiency. In addition, the feed conversion ratio (Figure 1B) significantly improved at *p* < 0.05, showing a better utilization of feed; a lower FCR value represents improved feed efficiency, i.e., less feed is required per unit of body weight gain. These results provide evidence for a positive effect of NaCl supplementation on the intake and growth performance of AOHU sheep in improving feed efficiency.

### 3.2. Faces and Urine Output, Nutrient Content, and Apparent Digestibility

NaCl supplementation significantly impacted the intake, excretion, and digestibility of nutrients in AOHU sheep (Table 2). A higher fecal (*p* < 0.05) and urine output (*p* < 0.01) were obtained in the NaCl group. The DMI was used in the computation of apparent nutrient digestibility. No differences were observed for fecal dry matter (DM), gross energy (GE), organic matter (OM), ether extract (EE), neutral detergent fiber (NDF), or acid detergent fiber (ADF) content (*p* > 0.05), although fecal crude protein (CP) content was lower in the NaCl group (*p* < 0.05). Particularly, NaCl supplementation significantly increased the digestibility of all the nutrients of major interest. The NaCl group exhibited a greater digestibility of DM, OM, EE, GE, CP, NDF, and ADF (all *p* < 0.01), and the CP digestibility was increased from 61.11 to 77.09%.

### 3.3. Carcass Yield, Dressing Percentage, Eye Muscle Area, Drip Loss, and Shear Force

**Figure 2:**
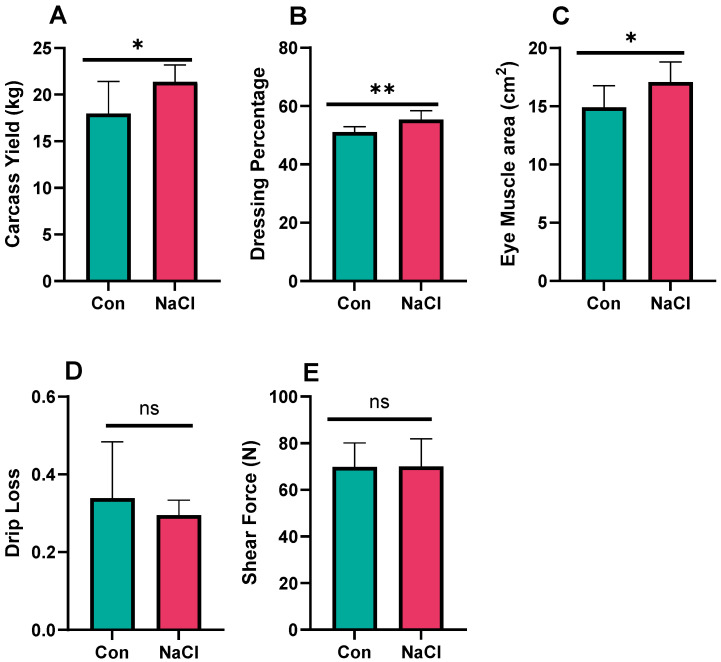
Effect of NaCl supplementation on meat yield and quality. (**A**) Carcass weight; (**B**) dressing percentage; (**C**) eye muscle area; (**D**) drip loss/moisture; (**E**) shear force/meat tenderness; Con = control; control diet contained 0.43% NaCl (DM basis); NaCl-supplemented diet contained 1.71% NaCl (DM basis); ** *p* < 0.01; * *p* < 0.05; ns = not significant (*p* > 0.05). Values are expressed as mean ± SEM, n = 8.

NaCl supplementation moderately enhanced carcass traits in AOHU sheep (Figure 2). Lambs treated with NaCl produced a greater carcass yield (Figure 2A; *p* < 0.05), greater dressing percentage (Figure 2B; *p* < 0.01), and larger Eye muscle area (EMA) of the Longissimus thoracis (Figure 2C; *p* < 0.05), indicating greater muscling and carcass development. Drip loss and shear force were not significantly affected (*p* > 0.05).

### 3.4. Meat pH and Color

**Figure 3:**
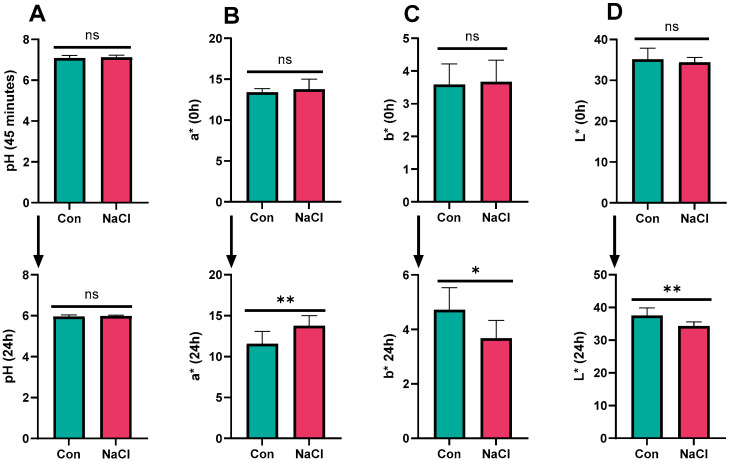
Effect of NaCl supplementation on meat pH and color. (**A**) Meat pH; (**B**) meat redness; (**C**) meat yellowness; (**D**) meat lightness; Con = control; control diet contained 0.43% NaCl (DM basis); NaCl-supplemented diet contained 1.71% NaCl (DM basis); ** *p* < 0.01; * *p* < 0.05; ns = not significant (*p* > 0.05). Values are expressed as mean ± SEM, n = 8.

Dietary supplementation of NaCl had no apparent effect on postmortem muscle pH decline, as pH values at 45 min and 24 h did not differ between the NaCl and control groups (Figure 3). The 24 h NaCl group had a greater a* (Figure 3B; *p* < 0.01) value, while the control group had greater b* (Figure 3C; *p* < 0.05) and L* (Figure 3D; *p* < 0.01) values.

### 3.5. Meat Muscle Amino Acid Profiles

The amino acid composition of the longissimus dorsi muscle for the control and NaCl-supplemented groups is presented in Table 3. Overall, NaCl supplementation had a moderate impact on the essential amino acids (EAAs), while non-essential amino acids (NEAAs) were relatively consistent. Specifically, the concentrations of threonine (*p* = 0.01), valine (*p* = 0.03), methionine (*p* = 0.01), isoleucine (*p* = 0.04), leucine (*p* = 0.001), and lysine (*p* = 0.01) were all higher in the NaCl-treated group compared to the control. Aspartic acid, glutamic acid, glycine, alanine, serine, tyrosine, phenylalanine, histidine, arginine, and proline, however, did not (*p* > 0.05) differ among treatments.

### 3.6. Meat Muscle Fatty Acid Profiles

The fatty acid composition of the longissimus dorsi muscle is summarized in Table 4. NaCl supplementation significantly (*p* < 0.05) decreased the concentration of C20:2 (cis-11,14-eicosadienoic acid), C20:3n6 (dihomo-γ-linolenic acid), and C22:0 (docosanoic acid) compared with the control group. Although these long-chain PUFA components were reduced, as reflected by the improved fatty acid profile, the PUFA/SFA value became higher (0.163 vs. 0.135) and the total SFA content lower in the NaCl-supplemented group than in the control animals (968.09 vs. 1270.50 mg/100 g).

## 4. Discussion

### 4.1. Intake, Growth Performance, and Feed Efficiency

Improvements in growth performance noted in the NaCl-supplemented group could be related to the higher dry matter intake and water intake measured, probably supporting greater nutrient availability. While sodium is well recognized for its role in osmoregulation and can stimulate thirst and feeding [17,18,19,20,21]. These physiological mechanisms are not directly assessed in the present study; thus, results should be interpreted in terms of the intake responses observed rather than a sodium-specific stimulatory effect.

Although water intake was apparently higher for the NaCl-supplemented group, these measurements were taken at the group level without replication and so cannot be statistically analyzed. Thus, no mechanistic inferences can be made from these observations. However, it is known from other studies that elevated dietary sodium increases water intake because of changes in osmotic balance that can have effects on rumen function and dynamics of digesta flow [22,23,24]. Those known mechanisms are mentioned here only for background and not as evidence from the current study. Thereby, a chain of physiological responses leads to increased DMI, increased ADG, and improved feed conversion efficiency. These performance improvements are in agreement with previous reports in sheep, goats, and cattle, in which moderate additions of salt were shown to improve digestibility and metabolic efficiency [25,26]. Additionally, in our earlier study under similar conditions, NaCl supplementation improved total energy intake [12], indicating that both increased intake and better nutrient use may also play a role in the improved growth performance seen in this experiment.

### 4.2. Faces and Urine Output, Nutrient Content, and Apparent Digestibility

There is increased intake of salt, which increases the blood osmotic pressure, thus triggering animals to increase water intake in order to maintain electrolytes. This osmotic challenge, through stimulation of thirst and diuresis, leads to the production of more urine (polyuria) [20,27], as indirectly observed by wetter bedding in the NaCl group. Increased water intake also contributes to increased gut hydration, promoting optimal rumen fermentation and microbial activity and digesta flow, hence increased digestion and absorption of nutrients [28].

Accordingly, the basal diets were formulated to be as similar as possible in macronutrient composition and energy content, while the NaCl-supplemented diet only differed to simulate saline–alkaline feeding conditions. However, the NaCl group had significantly higher apparent digestibility for all investigated nutrients: DM, OM, GE, CP, NDF, ADF (*p* < 0.001), and EE (*p* < 0.003). These increases indicate that NaCl-enriched alfalfa-based TMR may have a positive impact on microbial growth and fermentation efficiency. The higher DM and OM digestibility suggests better feed breakdown and nutrient availability. The higher digestibility of CP may thus indicate a more effective protein metabolism, probably due to enhanced microbial protein synthesis, improving amino acid absorption [29,30,31]. Similarly, the increased digestibility of EE suggests better utilization of lipids, which agrees with earlier studies where it was indicated that NaCl supplementation at a moderate level can improve fat digestion [29].

The 20% increase in the digestibility of fiber (NDF and ADF) in the NaCl-supplemented group is especially significant, as fiber is a substantial source of energy in ruminants. Although the underlying microbial mechanisms were not evaluated in this study, the increase in digestibility may reflect an overall improvement in nutrient use associated with NaCl supplementation. Furthermore, the higher fecal output and reduced fecal dryness in the NaCl group are consistent with other studies in which water excretion was increased in response to high salt intake [32,33,34]. Increased urine volume in animals fed high dietary salt is also consistent with known renal responses that maintain osmotic balance and electrolyte excretion [35,36].

In summary, the concurrent enhancement of water and feed intake under NaCl-supplemented feeding conditions imitating saline–alkaline conditions augmented nutrient digestibility and utilization of nitrogen and led to improved physiological performance and production efficiency of AOHU sheep.

### 4.3. Carcass Yield, Dressing Percentage, Eye Muscle Area, Drip Loss, and Shear Force

The improvements in carcass traits observed in the AOHU sheep in this experiment reflect the collective effects of the greater nutrient intake and digestibility recorded in the NaCl-supplemented group of animals. These findings differ from previous studies involving Santa Inês lambs, in which NaCl supplementation failed to change the percentage or yield of the carcasses [37]. Differences between studies could be related to breed differences, basal diet composition, and environmental conditions rather than to a direct, breed-specific adaptation to NaCl.

Further methodological differences explain the differing results in supplementation dosage, duration, and management practices, underlining the requirement for breed-specific nutritional strategies. Although the growth benefits were realized, studies always fail to report any differences regarding meat tenderness and water-holding capacity by NaCl supplementation. For instance, lambs that received salt-supplemented water had no adverse effect on meat quality traits [31,38].

Collectively, these findings imply that moderate NaCl supplementation of alfalfa-based TMR formulated to mimic saline–alkaline feeding regimes may be a viable nutrition plan for improving growth efficiency and carcass quality in semi-arid or saline-habituated sheep. The plan ensures nutrient digestibility and metabolic balance, giving insights to optimize feed formulation and sustainable ruminant production systems in harsh environments.

### 4.4. Meat pH and Color

The 24 h NaCl group had higher a* (*p* < 0.01), while the control group exhibited higher b* (*p* < 0.05) and L* values (*p* < 0.01) (Figure 3). Redness (a*) is indicative of myoglobin content and is normally associated with freshness and desirability of meat [39]. Yellowness (b*) and lightness (L*) define the fat-related coloration and general brightness of meat, respectively; both factors influence visual appeal and consumer acceptance [40]. Light meat (with higher L* values) is commonly judged to be more tender and visually attractive to customers and thus more acceptable (as products). Moreover, yellowness is related to the deposition of carotenoids, which is also perceived by consumers as an indicator of nutritional value [41]. Greater redness in the NaCl-supplemented group, together with increased lightness and yellowness in the control group, may be linked to NaCl-induced differences in muscle water-holding capacity and the stability of myoglobin.

Sodium can cause changes in the intracellular osmotic balance and alter protein interactions. The increased redness (a*) in NaCl-supplemented lamb meat is linked to enhanced consumer perceptions of freshness and quality. Instrumental color studies show that consumers begin to judge lamb as “acceptable” when a* ≥ 9.5, while 95% of consumers require a* ≥ 14.5 [42]. Conversely, the control group could be perceived as less fresh because of a larger value of lightness and yellowness, which may lower the rate of consumer acceptance [43,44].

### 4.5. Meat Muscle Amino Acid Profiles

NaCl-added group of this trial had greater threonine, valine, methionine, isoleucine, leucine, and lysine—growth factors directly involved in muscle growth, tissue repair, and meat flavor formation [45,46]. This could be attributed to the role of sodium in supporting nutrient utilization, osmotic balance, and transport of amino acids across cell membranes and hence increasing greater use of dietary protein. Sodium also may improve rumen microbial function as well as nitrogen metabolism, which are procedures that allow for increased synthesis and availability of amino acids for deposition into muscle [47]. Furthermore, optimal sodium composition can also be stated to stimulate feed consumption and increase rumen fermentation efficiency, leading to increased microbial protein production and improved availability of amino acids to the host animal [12,48].

Collectively, these findings indicate that supplementation with NaCl—applied here in order to mimic the salt levels characteristic of alfalfa grown under high saline–alkaline conditions—will improve protein accretion and maintain meat amino acid integrity, thus enhancing the nutritional quality of alfalfa-based sheep feed in marginal environments.

### 4.6. Meat Muscle Fatty Acid Profiles

The long-chain polyunsaturated fatty acids—C20:2 (cis-11, 14-eicosadienoic acid) and C20:3n6 (dihomo-γ-linolenic acid)—are reduced in the NaCl group. Although this may reduce the anti-inflammatory potential to a certain degree, the overall fat profile improved. Salt-mediated changes in rumen fermentation include elevated liquid fill and altered VFA production and pH, while some studies have also demonstrated that NaCl can improve microbial protein synthesis in spite of sometimes depressed organic matter digestibility [49,50]. The PUFA/SFA ratio increased (0.135 vs. 0.163), and thus, a leaner and healthier cardiac fat profile. Such an increase in the ratio of polyunsaturated to saturated fatty acids is associated with a reduced prevalence of cardiovascular conditions because it tends to result in lower LDL cholesterol concentrations [51]. The amount of saturated fatty acid C22:0 (docosanoic acid) was lower in the NaCl group. A reduction in total SFA (1270.50 vs. 968.09 mg/100 g) and specific SFAs such as C22:0 (behenic acid) in the NaCl group also enhances the nutritional quality of the meat, making it attractive to health-oriented consumers [52]. These changes may be because of the shift in rumen microbiota, as it is known that high salt intake alters microbial populations [53], thereby affecting the pathways of fatty acid biohydrogenation [54]. The disruptions of electrolyte balance due to increased sodium intake may also have an effect on lipid transport and tissue metabolism.

These findings together indicate that providing the saline-alkaline feeding conditions by NaCl-supplemented alfalfa-based TMR supports better growth and feed utilization in addition to improving health-related lipid profiles of sheep meat. This finding underlines the adaptive metabolic capacity of AOHU sheep to saline environments and further confirms the potential of NaCl supplementation at a moderate level as a nutritional strategy in improving productivity and quality in salt-affected regions.

## 5. Conclusions

The supplementation of NaCl to an alfalfa-based TMR increased lamb growth performance, feed conversion efficiency, and nutrient digestibility, along with a moderate increase in carcass yield, in the present experiment. Besides that, it also improved fat quality, reflected in a higher PUFA/SFA ratio and reduced level of C22:0. Furthermore, NaCl supplementation also improved the essential amino acid profile, particularly threonine, valine, methionine, isoleucine, leucine, and lysine, suggesting an improvement in muscle nutritional quality. These findings should be interpreted in light of key limitations, including a small number of animals were used in the experiment, there was no replication for the measurement of water intake, and the saline–alkaline feeding condition was simulated using only NaCl, which by no means represents the complexity of natural saline–alkaline forage. The inclusion of salt-tolerant or saline-affected forages in TMR has the potential to confer nutritional benefits to sheep under conditions that are marginal or prone to salinity, while further research is required for testing multiple levels of NaCl, measuring microbial responses directly, and investigating highly saline-affected forages for practical feeding strategies. Such a study will be helpful for optimizing forage utilization under saline conditions for added sustainability in livestock production.

## Figures and Tables

**Table 1:** Proportions and nutrient contents of test diets (%, dry matter basis). Adapted from our previous study [12].

Ingredients	Proportions	
	Control	NaCl
Cornmeal	46.50	46.50
Cottonseed meal	8.00	8.00
Wheat bran	8.00	8.00
Corncob meal	5.00	5.00
Alfalfa hay	30.00	30.00
Limestone	1.00	1.00
Salt	0.50	2.00
Premix ^1^	1.00	1.00
Total	100	100
Nutritional composition		
DM (air dry basis)	85.41	85.41
CP	15.34	15.34
GE, MJ/kg	18.15	18.15
ME, MJ/kg	10.2	10.2
EE	4.14	4.14
NDF	36.7	36.7
ADF	21.4	21.4
Ash	1.14	1.14
Ca	0.47	0.47
P	0.29	0.29
NaCl	0.43	1.71

^1^: Each kilogram of diet provided: VA 15,000 IU; VD 2200 IU; VE 50 IU; Fe 55 mg; Mn 47 mg; Zn 24 mg; Cu 12.5 mg; Se 0.5 mg; I 5 mg; and Co 0.1 mg. Abbreviations: DM, dry matter; CP, crude protein; GE, gross energy; ME, metabolizable energy; EE, ether extract; NDF, neutral detergent fiber; ADF, acid detergent fiber; Ca, calcium; P, phosphorus; MJ/kg, megajoules per kilogram; VA, vitamin A; VD, vitamin D; VE, vitamin E; Fe, iron; Mn, manganese; Zn, zinc; Cu, copper; Se, selenium; I, iodine; Co, cobalt; mg, milligram. Control diet contained 0.43% NaCl (DM basis); NaCl-supplemented diet contained 1.71% NaCl.

**Table 2:** Fecal and urine output, faces nutrient content, and apparent digestibility of nutrients.

Items	Groups			
	Control	NaCl	SEM	*p*-Value
Output				
Fecal output (kg/d)	0.90 ^b^	1.24 ^a^	0.14	<0.038
Urine output (mL/d)	751.00 ^b^	1733.71 ^a^	311.46	<0.01
Faces nutrient content (%)				
DM	44.25 ^a^	36.89 ^a^	3.827	0.083
GE (MJ/kg)	17.54 ^a^	17.59 ^a^	0.19	0.8
OM	93.06 ^a^	93.67 ^a^	0.646	1.29
EE	4.00 ^a^	3.64 ^a^	0.30	0.258
CP	17.58 ^a^	15.54 ^b^	0.68	<0.014
NDF	59.31 ^a^	57.90 ^a^	0.683	3.36
ADF	36.98 ^a^	37.47 ^a^	0.798	1.88
Faces nutrient digestibility (%)				
DM	65.97 ^b^	77.43 ^a^	2.09	<0.001
OM	67.98 ^b^	78.59 ^a^	2.05	<0.001
EE	66.89 ^b^	80.23 ^a^	3.59	<0.003
GE	67.10 ^b^	78.13 ^a^	2.12	<0.001
CP	61.11 ^b^	77.09 ^a^	2.19	<0.001
NDF	45.13 ^b^	64.44 ^a^	3.69	<0.001
ADF	41.38 ^b^	60.52 ^a^	4.07	<0.001

Different superscript letters indicate a significant difference (*p* < 0.05). DM: dry matter; OM: organic matter; GE: gross energy; EE: ether extract; CP: crude protein; NDF: neutral detergent fiber; ADF; acid detergent fiber. Control diet contained 0.43% NaCl (DM basis); NaCl-supplemented diet contained 1.71% NaCl (DM basis); Values are expressed as mean ± SEM, n = 8.

**Table 3:** Meat muscle amino acid profiles.

Amino Acids (mg/100 g)	Groups		SEM	*p*-Value
	Con	NaCl		
Aspartic acid	17.36	17.12	0.28	0.69
Threonine	8.54 ^b^	9.42 ^a^	0.20	0.01
Serine	7.35	7.28	0.14	0.82
Glutamic acid	29.64	28.89	0.55	0.52
Glycine	8.41	8.58	0.16	0.63
Alanine	11.01	11.15	0.16	0.68
Cysteine	1.57	1.40	0.06	0.20
Valine	7.35 ^b^	7.99 ^a^	0.35	0.03
Methionine	3.81 ^b^	4.56 ^a^	0.12	0.01
Isoleucine	6.61 ^b^	7.27 ^a^	0.33	0.04
Leucine	14.70 ^b^	15.59 ^a^	0.33	0.001
Tyrosine	6.49	6.33	0.15	0.64
Phenylalanine	6.78	6.73	0.23	0.93
Histidine	8.62	8.93	0.14	0.27
Lysine	15.86 ^b^	16.51 ^a^	0.36	0.01
Arginine	10.97	10.77	0.25	0.72
Proline	6.75	7.01	0.14	0.38

Different superscript letters indicate a significant difference (*p* < 0.05). Values are presented as mean ± SEM (n = 8). Control diet contained 0.43% NaCl (DM basis); NaCl-supplemented diet contained 1.71% NaCl (DM basis); SEM: standard error of the mean.

**Table 4:** Fatty acid composition of Longissimus thoracis muscle (mg/100 g muscle, mean ± SEM).

Fatty Acids	Groups		SEM	*p*-Value
	Con	NaCl		
C10:0	1.76	0.62	0.36	0.11
C12:0	0.70	0.86	0.21	0.73
C14:0	51.71	38.64	5.56	0.26
C14:1	1.75	1.18	0.20	0.16
C15:0	9.06	7.27	1.01	0.40
C16:0	651.38	474.1	50.3	0.08
C16:1	52.81	40.90	3.88	0.13
C17:0	32.69	25.88	2.80	0.24
C18:0	513.60	413.0	42.4	0.25
C18:1n9c- C18:1c9	1059.7	805.84	76.47	0.10
C18:2n6c	106.79	98.26	3.79	0.28
C20:0	2.91	1.92	0.33	0.14
C18:3n6γ	1.98	1.57	0.14	0.15
C18:3n3α	6.34	5.83	0.35	0.50
C20:1	3.40	3.07	0.15	0.30
C20:2	1.88 ^a^	1.16 ^b^	0.16	0.02
C22:0	1.49 ^a^	0.91 ^b^	0.14	0.03
C20:3n6	3.80 ^a^	3.28 ^b^	0.13	0.03
C20:4n6	46.45	43.59	1.25	0.27
C23:0	2.73	2.74	0.16	0.97
C20:5n3	1.52	1.30	0.14	0.46
C24:0	2.47	2.15	0.17	0.39
C24:1	4.00	3.24	0.45	0.43
C22:6n3	2.22	2.00	0.29	0.72
Total SFA	1270.50 ^a^	968.09 ^b^	35.2	0.04
Total PUFA	171.83	157.99	5.80	0.05
PUFA/SFA ratio	0.135 ^b^	0.163 ^a^	0.01	0.03

Different superscript letters indicate a significant difference (*p* < 0.05). Values are presented as mean ± SEM (n = 8). Control diet contained 0.43% NaCl (DM basis); NaCl-supplemented diet contained 1.71% NaCl (DM basis); SEM: standard error of the mean. SFA = saturated fatty acids; PUFA = Polyunsaturated fatty acids.

## Data Availability

The data presented in this study are available on request from the corresponding author due to ethical restrictions.

## References

[1] Chacko Kaitholil S.R., Mooney M.H., Aubry A., Rezwan F., Shirali M. (2024). Insights into the influence of diet and genetics on feed efficiency and meat production in sheep. Anim. Genet..

[2] Esen S. (2023). Optimizing ruminant nutrition: Insights from a comprehensive analysis of silage composition and in vitro gas production dynamics using nonlinear models. Biosystems.

[3] Zaman M., Shahid S.A., Heng L., Shahid S.A., Zaman M., Heng L. (2018). Soil salinity: Historical perspectives and a world overview of the problem. Guideline for Salinity Assessment, Mitigation and Adaptation Using Nuclear and Related Techniques.

[4] Chanu P.H. (2023). Management of Salt-Affected Soils for Increasing Crop Productivity. Enhancing Resilience of Dryland Agriculture Under Changing Climate: Interdisciplinary and Convergence Approaches.

[5] Guo R., Zhou Z., Cai R., Liu L., Wang R., Sun Y., Wang D., Yan Z., Guo C. (2024). Metabolomic and physiological analysis of alfalfa (*Medicago sativa* L.) in response to saline and alkaline stress. Plant Physiol. Biochem..

[6] Abebe H., Tu Y. (2024). Impact of salt and alkali stress on forage biomass yield, nutritive value, and animal growth performance: A comprehensive review. Grasses.

[7] Squires V.R. (1993). Australian experiences with high salinity diets for sheep. Towards the Rational Use of High Salinity Tolerant Plants.

[8] Pewan S.B., Otto J.R., Kinobe R.T., Adegboye O.A., Malau-Aduli A.E.O. (2020). MARGRA lamb eating quality and human health-promoting omega-3 long-chain polyunsaturated fatty acid profiles of Tattykeel Australian White Sheep: Linebreeding and gender effects. Antioxidants.

[9] Yuan J.-D., Wang L.-W., Fu S.-Y., E R.-G.-L.-T., Ren X.-Q., Sun H., Liu F., Wang B., An J.-H., Zhao M.-R. (2025). Heat Tolerance Differences Between Hu Sheep and Hu Crossbred Sheep in Microbial Community Structure and Metabolism. Metabolites.

[10] Li S., Bao Y., Lv M., Zhang L., Liu L., Liu Y., Lu Q. (2023). Comparative Na+ and K+ profiling reveals microbial community assembly of alfalfa silage in different saline-alkali soils. Fermentation.

[11] China Legal Publishing House (2007). Nutrient Requirements of Small Ruminants: Sheep, Goats, Cervids, and New World Camelids.

[12] Abebe H., Yang R., Wei G., Feng X., Tu Y. (2025). Feeding with a NaCl-Supplemented Alfalfa-Based TMR Improves Nutrient Utilization, Rumen Fermentation, and Antioxidant Enzyme Activity in AOHU Sheep: A Nutritional Simulation of Saline–Alkaline Conditions. Fermentation.

[13] Thiex N. (2009). Evaluation of analytical methods for the determination of moisture, crude protein, crude fat, and crude fiber in distillers dried grains with solubles. J. AOAC Int..

[14] Van Soest P.v., Robertson J.B., Lewis B.A. (1991). Methods for dietary fiber, neutral detergent fiber, and nonstarch polysaccharides in relation to animal nutrition. J. Dairy Sci..

[15] Zhang S., Xie Y., Li M., Yang H., Li S., Li J., Xu Q., Yang W., Jiang S. (2020). Effects of different selenium sources on meat quality and shelf life of fattening pigs. Animals.

[16] Wang K., Li L., Li N., Ke R., Yuan D., Deng T., Liu S., Wu Y., Zuo D., Fang H. (2023). The Assessment of Fatty Acid Composition in Deep-Fried Dough Sticks across Five Cities in China in 2020. Processes.

[17] Joyce J., Brunswick L. (1975). Sodium supplementation of sheep and cattle fed lucerne. N. Z. J. Exp. Agric..

[18] Mpendulo C., Chimonyo M., Zindove T. (2017). Influence of water restriction and salinity on feed intake and growth performance of Nguni does. Small Rumin. Res..

[19] Phillips C., Youssef M., Chiy P., Arney D. (1999). Sodium chloride supplements increase the salt appetite and reduce stereotypies in confined cattle. Anim. Sci..

[20] Zoidis E., Hadjigeorgiou I. (2017). Effects of drinking saline water on food and water intake, blood and urine electrolytes and biochemical and haematological parameters in goats: A preliminary study. Anim. Prod. Sci..

[21] Goatcher W., Church D. (1970). Taste responses in ruminants. III. Reactions of pygmy goats, normal goats, sheep and cattle to sucrose and sodium chloride. J. Anim. Sci..

[22] Nation H.L., Simmonds S.S., Stocker S.D. (2016). Brain Epithelial Sodium Channel Contributes to Thirst Stimulated by Hypernatremia. FASEB J..

[23] Ramadhan M.R., Schlecht E., Dickhöfer U., Mahgoub O., Jörgensen R.G. (2022). Feed digestibility, digesta passage and faecal microbial biomass in desert-adapted goats exposed to mild water restriction. J. Anim. Physiol. Anim. Nutr..

[24] Bujňák L., Maskaľová I., Vajda V. (2011). Determination of buffering capacity of selected fermented feedstuffs and the effect of dietary acid-base status on ruminal fluid pH. Acta Vet. Brno.

[25] Champness M., McCormick J., Bhanugopan M., McGrath S. (2020). Sodium deficiency in lucerne (*Medicago sativa*) forage in southern Australia and the effect of sodium and barley supplementation on the growth rate of lambs grazing lucerne. Anim. Prod. Sci..

[26] McClymont G., Wynne K., Briggs P., Franklin M. (1957). Sodium chloride supplementation of high-grain diets for fattening Merino sheep. Aust. J. Agric. Res..

[27] Visscher C., Witzmann S., Beyerbach M., Kamphues J. (2013). Watering cattle (young bulls) with brackish water–a hazard due to its salt content?. Tierärztl. Prax. Ausg. G Großtiere/Nutztiere.

[28] Hoover W.H., Miller T.K. (1991). Rumen digestive physiology and microbial ecology. Vet. Clin. N. Am. Food Anim. Pract..

[29] Oliveira F.M.d., Oliveira G.J.C.d., Oliveira M.L.A.d., Jaeger S.M.P.L., Almeida L.H.S., Nery I.B.Q., Leite L.C. (2016). Consumo e digestibilidade de nutrientes em ovinos alimentados com sal forrageiro de faveleira (*Cnidoscolus phyllacanthus*). Rev. Bras. Saúde Prod. Anim..

[30] Mahan D., Wiseman T., Weaver E., Russell L. (1999). Effect of supplemental sodium chloride and hydrochloric acid added to initial starter diets containing spray-dried blood plasma and lactose on resulting performance and nitrogen digestibility of 3-week-old weaned pigs. J. Anim. Sci..

[31] Yirga H., Urge M., Goetsch A.L., Tolera A., Puchala R., Patra A.K. (2024). Effects of Salinity Levels of Drinking Water on Water Intake and Loss, Feed Utilization, Body Weight, Thermoregulatory Traits, and Blood Constituents in Growing and Mature Blackhead Ogaden Sheep and Somali Goats. Animals.

[32] Moseley G., Jones D. (1974). The effect of sodium chloride supplementation of a sodium adequate hay on digestion, production and mineral nutrition in sheep. J. Agric. Sci..

[33] Kii W.Y., Dryden G.M. (2005). Effect of drinking saline water on food and water intake, food digestibility, and nitrogen and mineral balances of rusa deer stags (*Cervus timorensis* russa). Anim. Sci..

[34] Phillips C., Mohamed M., Omed H. (2003). The effects of increasing the sodium content of grass or concentrates on the nutrition of sheep. Anim. Sci..

[35] Thiet N., Van Hon N., Ngu N.T., Thammacharoen S. (2022). Effects of high salinity in drinking water on behaviors, growth, and renal electrolyte excretion in crossbred Boer goats under tropical conditions. Vet. World.

[36] Nguyen T., Van Truong K., Nguyen K.K.T., Nguyen N.T., Chaiyabutr N., Thammacharoen S. (2024). Effects of diluted seawater in drinking water on physiological responses, feeding, drinking patterns, and water balance in crossbred dairy goats. Vet. World.

[37] Pereira Filho J.M., Silva A.M., Silva D.S., Cézar M.F., Bezerra L.R., Rufino S.R., Borburema J.B., Bayão G.F. (2014). Carcass characteristics of Santa Inês lambs finished on native pasture and subjected to different types of supplementation. Rev. Ciênc. Agrár..

[38] Yousfi I., Salem H.B., Aouadi D., Abidi S. (2016). Effect of sodium chloride, sodium sulfate or sodium nitrite in drinking water on intake, digestion, growth rate, carcass traits and meat quality of Barbarine lamb. Small Rumin. Res..

[39] Ma P., Yin J., Sun Y., Wu D., Zhang Y., Feng Y., Liu G. (2024). New insights into NO bonding in Tan sheep myoglobin for meat pigmentation: Spectroscopic and density functional theory investigations. J. Mol. Struct..

[40] Sun J., Yang X., Zhao G., He Z., Xing W., Chen Y., Tan X., Wang M., Li W., An B. (2024). Protein phosphatase 1 catalytic subunit gamma is a causative gene for meat lightness and redness. PLoS Genet..

[41] Han J., Wang Y., Wang Y., Hao S., Zhang K., Tian J., Jin Y. (2024). Effect of changes in the structure of myoglobin on the color of meat products. Food Mater. Res..

[42] Khliji S., Van de Ven R., Lamb T., Lanza M., Hopkins D. (2010). Relationship between consumer ranking of lamb colour and objective measures of colour. Meat Sci..

[43] Corlett M.T., Pethick D.W., Kelman K.R., Jacob R.H., Gardner G.E. (2021). Consumer perceptions of meat redness were strongly influenced by storage and display times. Foods.

[44] Tabibian S., Mohsenzadeh M., Pourreza H., Golzarian M. (2017). Sensory evaluation of the color of mutton by computer vision system. Iran. J. Vet. Sci. Technol..

[45] Duran B.O.S., Zanella B.T.T., Perez E.S., Mareco E.A., Blasco J., Dal-Pai-Silva M., Garcia de la Serrana D. (2022). Amino acids and IGF1 regulation of fish muscle growth revealed by transcriptome and microRNAome integrative analyses of pacu (*Piaractus mesopotamicus*) myotubes. Int. J. Mol. Sci..

[46] Rai P. (2023). Role of essential amino acids in protein synthesis and muscle growth. J. Biochem. Res..

[47] Ma X., Yu M., Liu Z., Deng D., Cui Y., Tian Z., Wang G. (2020). Effect of amino acids and their derivatives on meat quality of finishing pigs. J. Food Sci. Technol..

[48] Churchward-Venne T.A., Murphy C.H., Longland T.M., Phillips S.M. (2013). Role of protein and amino acids in promoting lean mass accretion with resistance exercise and attenuating lean mass loss during energy deficit in humans. Amino Acids.

[49] White H.C., Davis N.G., Van Emon M.L., DelCurto-Wyffels H.M., Wyffels S.A., DelCurto T. (2024). Impacts of increasing levels of salt on intake, digestion, and rumen fermentation with beef cattle consuming low-quality forages. J. Anim. Sci..

[50] Godwin I.R., Williams V.J. (1986). Effects of intraruminal sodium chloride infusion on rumen and renal nitrogen and electrolyte dynamics in sheep. Br. J. Nutr..

[51] Zhao Y., Xie B., Gao J., Zhao G. (2020). Dietary supplementation with sodium sulfate improves rumen fermentation, fiber digestibility, and the plasma metabolome through modulation of rumen bacterial communities in steers. Appl. Environ. Microbiol..

[52] Richard D., Bausero P., Schneider C., Visioli F. (2009). Polyunsaturated fatty acids and cardiovascular disease. Cell. Mol. Life Sci..

[53] Hammad S., Pu S., Jones P.J. (2016). Current evidence supporting the link between dietary fatty acids and cardiovascular disease. Lipids.

[54] Chen Y., Cheng Z., Duan Q., Meng Q., Tao X., Shang B., Dong H. (2017). Age-related response of rumen microbiota to mineral salt and effects of their interactions on enteric methane emissions in cattle. Microb. Ecol..

